# Effects of Dorsal or Ventral Predominant Frontotemporal Neurodegeneration on Interval Prosodic Key Variation

**DOI:** 10.1002/alz70857_103542

**Published:** 2025-12-25

**Authors:** Peter S. Pressman, Francesca R. Dino, Peter Foltz, Robert W Levenson

**Affiliations:** ^1^ University of Colorado School of Medicine, Aurora, CO, USA; ^2^ Layton Healthy Aging and Alzheimer's Disease Research Center, Portland, OR, USA; ^3^ University of Colorado, Anschutz Medical Campus, Aurora, CO, USA; ^4^ Institute of Cognitive Science, University of Colorado, Boulder, Boulder, CO, USA; ^5^ University of California, Berkeley, Berkeley, CA, USA

## Abstract

**Background:**

Prosodic deficits in neurodegenerative diseases are often studied through a left‐right hemispheric framework, particularly focusing on pitch control. Emerging evidence, however, suggests prosodic processing may also follow dorsal‐ventral organizational principles tied to different time windows of signal variation. For example, most studies of pitch consider variation overall, and have tied this to frontal pathways. However, speakers can also change median pitch, or key, between intervals of speech to signify a change of context or emphasis, which requires semantic knowledge usually mediated by ventral pathways. This study explored pitch variation across conversational speech intervals in individuals with behavioral variant frontotemporal dementia (bvFTD) and semantic variant primary progressive aphasia and semantic behavioral variant FTD (svPPA/sbvFTD), conditions primarily affecting dorsal and ventral pathways, respectively.

**Methods:**

We recorded ten‐minute semi‐structured conversations between 76 participants (31 bvFTD, 16 svPPA, 11 sbvFTD, 18 healthy controls) and study partners using lavalier microphones in the Berkeley Psychophysiology Laboratory. Uninterrupted speech intervals were manually labeled and analyzed in Praat. Python, assisted by Claude (AI), was used for statistical analyses. Pitch variability was quantified as the standard deviation of median interval pitch, reflecting slower‐scale prosodic modulation tied to conversational context shifts.

**Results:**

A between‐group ANOVA revealed marginal statistical insignificance (F=2.181, *p* = 0.098). However, pre‐specified analyses by pathway involvement showed that dorsal pathway disruption (FTD) was associated with significantly greater pitch variation compared to ventral pathway disruption (svPPA/rsvPPA) (t=2.351, *p* = 0.022, Cohen's d=0.629).

**Conclusion:**

These results suggest that prosodic key across conversational intervals is differentially impacted by dorsal versus ventral pathway degeneration. This supports theories of dorsal‐ventral distinctions in the temporal processing of speech and enhances our understanding of how distinct neural pathways regulate prosodic control within different time windows.

